# Downregulation of CacyBP by CRISPR/dCas9-KRAB Prevents Bladder Cancer Progression

**DOI:** 10.3389/fmolb.2021.692941

**Published:** 2021-06-11

**Authors:** Hanxiong Zheng, Chiheng Chen

**Affiliations:** Department of Urology, The Eighth Affiliated Hospital, Sun Yat-sen University, Shenzhen, China

**Keywords:** cacybp, bladder cacner, CRISPR, proliferation, migration

## Abstract

Bladder cancer (BCa) is a leading cause of cancer-related death in the world. CacyBP is initially described as a binding partner of calcyclin and has been shown to be involved in a wide range of cellular processes, including cell differentiation, proliferation, protein ubiquitination, cytoskeletal dynamics and tumorigenesis. In the present study, we found that CacyBP expression was significantly upregulated in BCa tissues compared with adjacent normal tissues. Moreover, its expression was negatively correlated with overall survival time. Secondly, CacyBP had higher expressions in BCa cell lines than normal urothelial cells which was consistent with the results of BCa tissues. Finally, knockdown of CacyBP by CRIPSR-dCas9-KRAB in T24 and 5,637 BCa cells inhibited cell proliferation and migration by CCK-8 assay and scratch assay, and promoted apoptosis by caspase-3/ELISA. These data elucidate that CacyBP is an important oncogene contributing to malignant behavior of BCa and provide a potentially molecular target for treatment of BCa.

## Introduction

Bladder cancer is the most common urinary tract tumor with a high degree of malignancy, most of which are urothelial carcinoma of the bladder, accounting for more than 90% (Pashos et al., 2002). Its incidence is high over the years, among all malignant tumors, it ranks the 10th in China (Chen et al., 2016). In 2012, the number of cases of bladder cancer in the world is expected to be as high as 430,000 (Feday et al., 2015). The morbidity and mortality of bladder cancer mainly occur in underdeveloped areas and developing countries, which will be significantly higher than that in developed areas (Antoni et al., 2017; Siegel et al., 2017). Bladder cancer is a heterogeneous disease with complex and varied clinical features and atypical early clinical symptoms. Most patients come to the hospital after hematuria occurs. As a result, when most patients are first diagnosed with bladder cancer, the tumor has already reached the middle and advanced stage, and most of the tumors have already undergone invasion and metastasis, leading to unsatisfactory treatment or recurrence of advanced bladder cancer (DeGeorge et al., 2017; Babjuk et al., 2017). Therefore, in order to explore the pathogenesis of bladder cancer, to analyze the molecular mechanism of its invasion and metastasis, and to find new screening biological indicators, diagnosis and treatment markers and molecular therapeutic targets for bladder cancer has always been a clinical difficulty and research hotspot. It is of great significance to improve the diagnosis and treatment effect of bladder cancer, reduce its recurrence rate and mortality rate, and improve the prognosis of patients.

CACYBP protein was first cloned from Eichenian ascites tumor cells by Filipek et al., with a molecular weight of about 30 kDa. It can bind to calcyclin, so it was first named as CACYCLIN binding protein (CACYBP) (Filipek and Wojda, 1996; Filipek et al., 2002; Nowotny et al., 2000). In 2001, Matsuzawa et al. studied the process of β-catenin degradation induced by p53 activation in tumor cells, and found a new SAIH-1 binding protein (SIP), which can bind with SAIH-1, SKPL, EBI, etc., to form a complex, and eventually ubiquitinate to degrade β-catenin (Matsuzawa and Reed, 2001). After amino acid sequence alignment, it was found that human SIP was highly homologous to mouse CacyBP, thus the naming of CacyBP/SIP protein was formally established. Currently, it is known that CacyBP/SIP plays a variety of molecular biological functions. It is not only involved in protein ubiquitin and proteasome degradation pathway, but also involved in the regulation of cell cycle, mediating apoptosis signal transduction, and even affecting the occurrence and development of tumors (olska—Wo§ et al., 2016; Ning et al., 2016).

Taking gastric cancer as an example, CacyBP/SIP can inhibit the proliferation of tumor cells, reduce the invasion ability, and prolong the survival of tumor-bearing mice (Ning et al., 2007). Another team studied the expression profile of CacyBP/SIP in 181 cases of gastric cancer. Although the expression of CacyBP/SIP was highly expressed in gastric cancer tissues, it was not correlated with clinicopathological characteristics (Zhai et al., 2015). In addition, besides high expression in gastric cancer, CacyBP/SIP is also highly expressed in pancreatic cancer (Zhai et al., 2008; Chen et al., 2008), and plays a role in promoting cancer progression (Chen X. et al., 2011). However, in renal cancer and chronic lymphoblastic leukemia, the expression of CacyBP/SIP is decreased (Sun et al., 2007; Fu et al., 2016), which plays a role in tumor inhibition. Interestingly, in breast cancer and glioma, the CacyBP/SIP expression profiles reported by different teams were completely opposite (Nie et al., 2010; Wang et al., 2010; Zhao et al., 2011; Shi et al., 2014). These results suggest that CacyBP/SIP may be involved in different molecular mechanisms in different tumors and play a complex biological function.

In our present study, we found that CacyBP expression was significantly upregulated in bladder cancer tissues compared with adjacent normal tissues. And its expression level is negatively correlated with overall survival time. Moreover, knockdown of CacyBP by CRISPR-dCas9-KRAB inhibited BCa cell proliferation and migration but promoted cells apoptosis. Taken together, these results provide novel insight into how CacyBP regulate bladder cancer tumorigenesis and provide a potential therapeutic target for bladder cancer.

## Results

### The Expression Level of CacyBP Was Significantly Upregulated in Bladder Cancer Tissues

**FIGURE 1:**
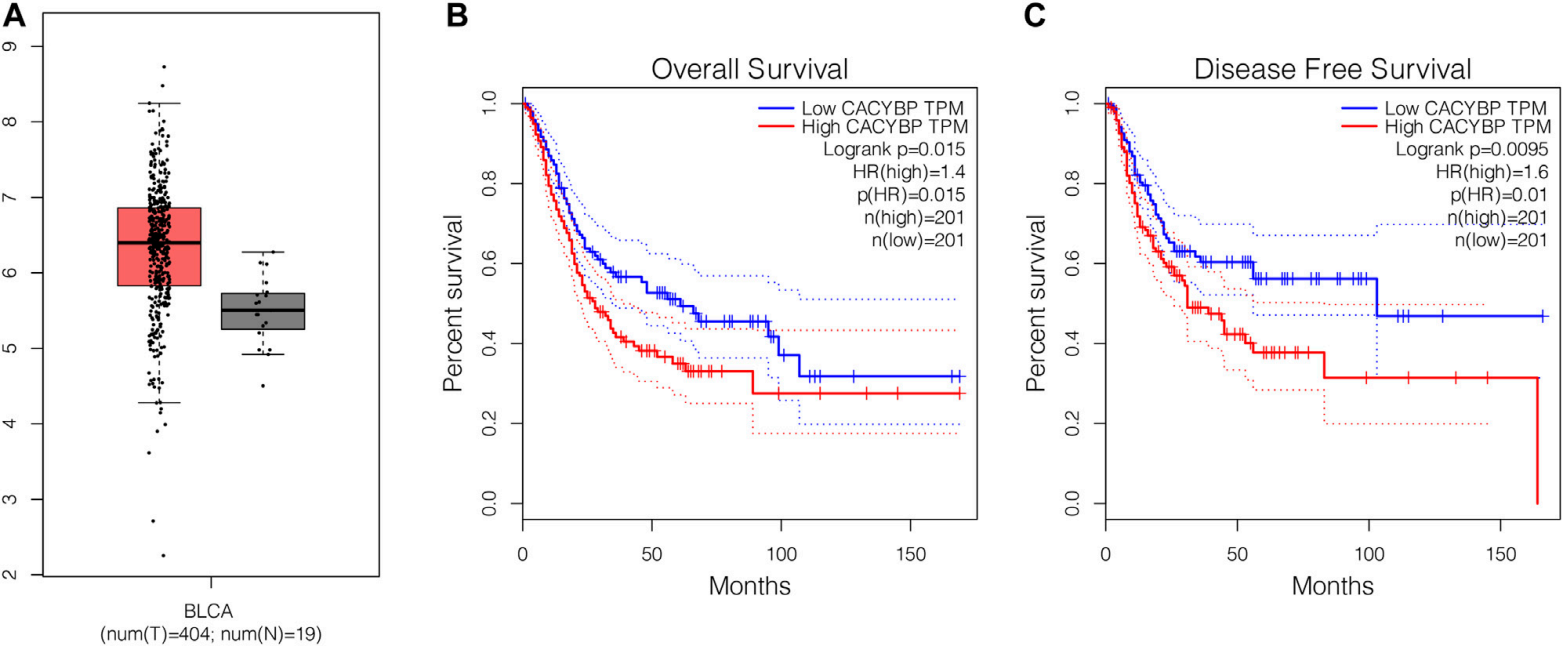
The mRNA level of CacyBP was significantly increased in bladder cancer tissues **(A)** CacyBP expression levels in tumor and adjacent tissues from TCGA database **(B)** The Kaplan–Meier survival curves of CacyBP in bladder cancer tissues from TCGA database **(C)** The disease-free survival curves of CacyBP in bladder cancer tissues from TCGA database.

To explore the expression patten of CacyBP in bladder cancer tissues, we firstly checked its expression level in TCGA database. The results showed that CacyBP was significantly upregulated in tumor tissues compared to normal tissues (Figure 1A). Furthermore, based on results of Kaplan–Meier survival curves, we found patients with high CacyBP expression level had a worse overall survival rate compared to those with low CacyBP expression (Figure 1B). In the disease-free survival analysis, we found the same results that patients with high CacyBP expression level had a worse survival rate compared to those with low CacyBP expression (Figure 1C). These results suggested that CacyBP was associated with the development and progression of bladder cancer.

### CRIPSR/dCas9-KRAB Decreased the Expression Level of CacyBP in Bladder Cancer Cells

**FIGURE 2:**
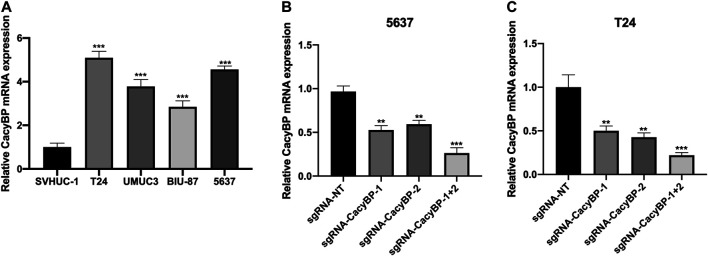
CRIPSR/dCas9-KRAB decreased the expression level of CacyBP in bladder cancer cells **(A)** The expression level of CacyBP in bladder cancer cell lines (T24, UMUC3, BIU-87 and 5,637) and normal urothelial cells (SVHUC-1) by RT-qPCR **(B)** The expression level of CacyBP in 5,637 cells after transfected with dCas9-KRAB and sgRNAs for 24 h by RT-qPCR **(C)** The expression level of CacyBP in T24 cells after transfected with dCas9-KRAB and sgRNAs for 24 h by RT-qPCR. **p < 0.01, ***p < 0.001.

We measured the expression level of CacyBP in bladder cancer cell lines and found it was upregulated in bladder cancer cell lines (T24, UMUC3, BIU-87 and 5,637) compared to normal urothelial cells (SVHUC-1) (Figure 2A). Previous study had confirmed that CRIPSR/dCas9-KRAB could decrease the mRNA level of targeted genes (Chavez et al., 2015). Then we transfected with dCas9-KRAB fusion protein in T24 and 5,637 cells, the results showed that the expression level of CacyBP was decreased when transfected with sgRNAs targeted CacyBP compared to sgRNA-NT (Figures 2B,C).

### Downregulation of CacyBP Inhibited the Proliferation of Bladder Cancer Cells

**FIGURE 3:**
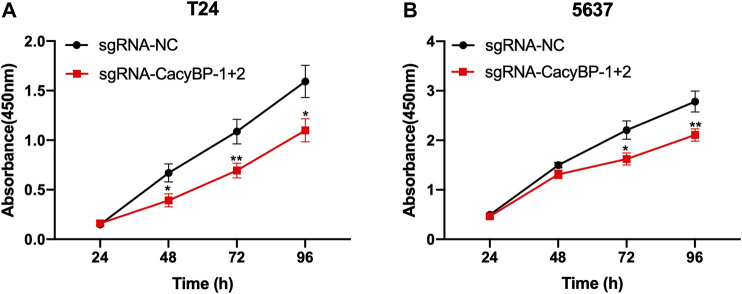
Downregulation of CacyBP inhibited the proliferation of bladder cancer cells **(A, B)** CCK-8 assays of T24 and 5,637 cells after transfected with dCas9-KRAB and sgRNAs. *p < 0.05, **p < 0.01.

Next, we determined whether the downregulation of CacyBP inhibited bladder cancer cells proliferation. We found that the downregulation of CacyBP by dCas9-KRAB in T24 and 5,637 cells significantly inhibited cell proliferation detected by CCK-8 assay (Figure 3A,B).

### Downregulation of CacyBP Inhibited the Migration of Bladder Cancer Cells

**FIGURE 4:**
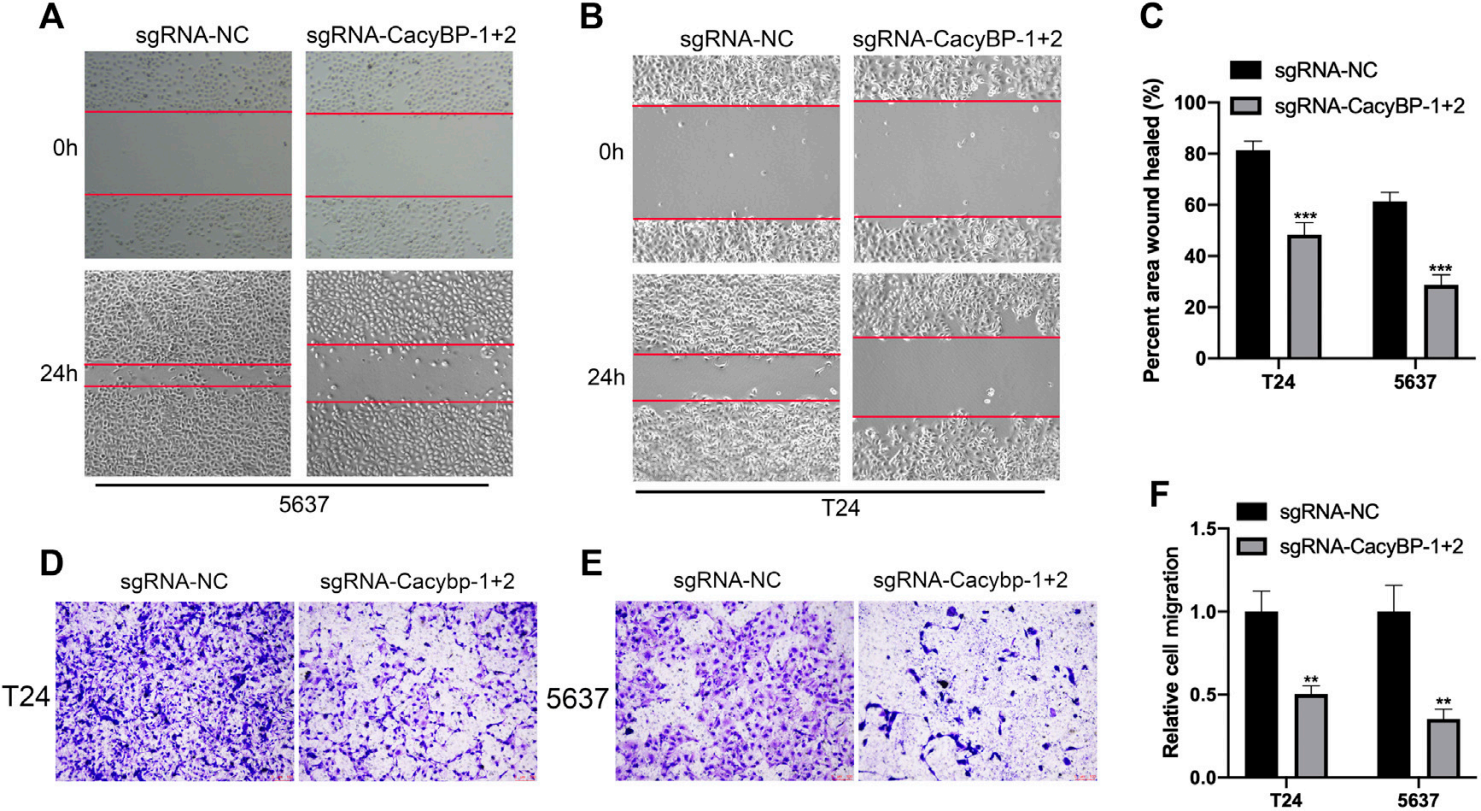
Downregulation of CacyBP inhibited the migration of bladder cancer cells **(A–C)** Wound-healing assays for migration ability of T24 and 5,637 cells after transfected with dCas9-KRAB and sgRNAs **(D–F)** Transwell assays for migration ability of T24 and 5,637 cells after transfected with dCas9-KRAB and sgRNAs. **p < 0.01, ***p < 0.001.

Besides, we further evaluated the migration ability after downregulated CacyBP in bladder cancer cell lines checked by wound healing and transwell migration assays. The wound-healing assays showed that CacyBP downregulated T24 and 5,637 cells closed the wound much slower than the controls after 24 h (Figures 4A–C). Transwell assays revealed that the migration ability of T24 and 5,637 cells was significantly decreased following the downregulation of CacyBP (Figures 4D–F). These results suggested that downregulated CacyBP by dCas9-KRAB inhibited the migration of bladder cancer cells.

### Downregulation of CacyBP Promoted the Apoptosis of Bladder Cancer Cells

**FIGURE 5:**
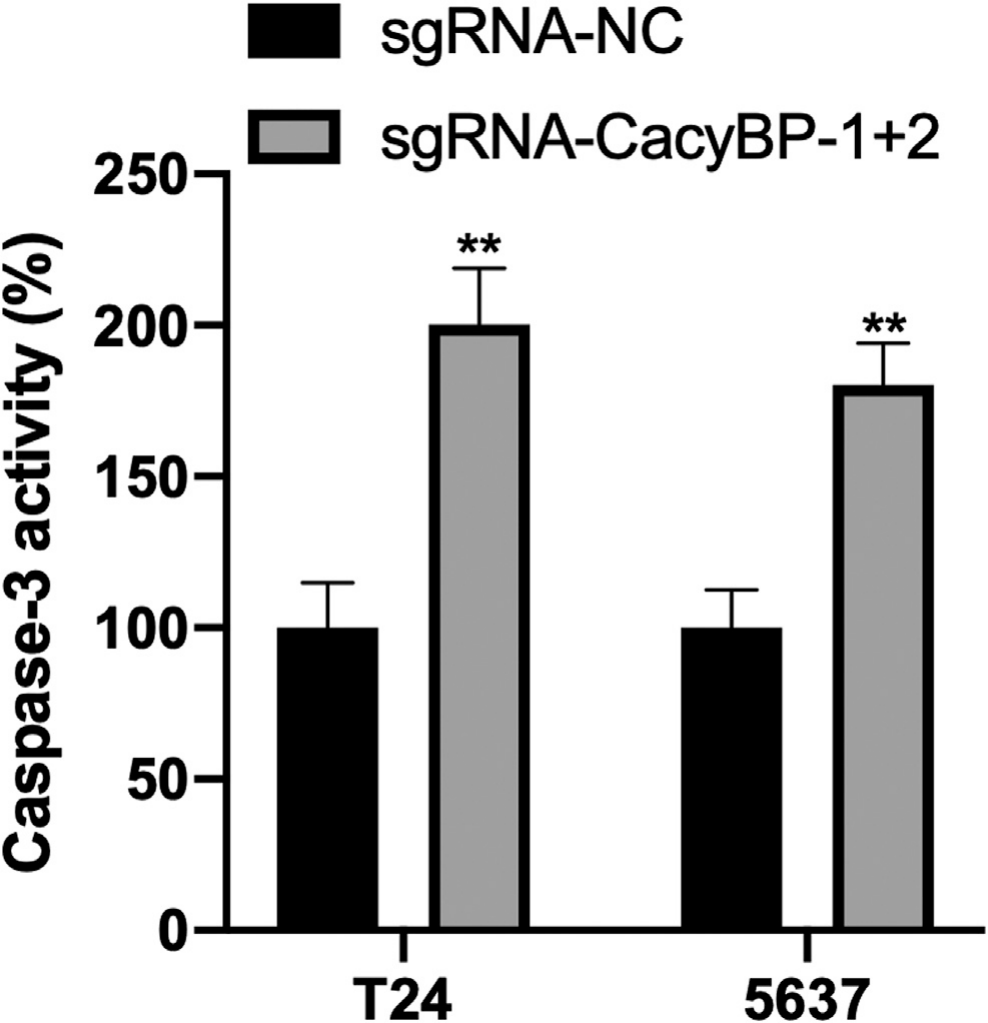
Downregulation of CacyBP promoted the apoptosis of bladder cancer cells detected by caspase3-Elisa. **p < 0.01.

Finally, we performed the caspase3-Elisa assay and found the CacyBP downregulated T24 and 5,637 cells significantly promoted the cells apoptosis compared to the controls (Figure 5fig5).

## Discussion

Bladder cancer is a common malignancy in genitourinary diseases and one of the ten most common human tumors. Bladder cancer is the second most common cancer in western countries, after prostate cancer (Siegel et al., 2014). Bladder cancer is characterized by high malignancy, multi-centrality, easy recurrence and progression, and long treatment time, easy to develop drug resistance, which seriously threatens human health (Smith et al., 2014). Therefore, it is an urgent scientific problem to actively explore the pathogenesis and development of bladder cancer as well as new therapeutic targets.

More and more studies have shown that CACYBP/SIP protein plays an important role in the occurrence, development, invasion and metastasis of various tumors. Therefore, the role and mechanism of CACYBP/SIP protein in tumors have become a research hotspot. In the past 10 years, people have studied the role of CACYBP/SIP in the occurrence and development of a variety of tumors. For instance, the high expression of CACYBP/SIP in pancreatic cancer cells is positively correlated with different stages and distant metastasis, suggesting that the high expression of CACYBP/SIP may be associated with the aggressiveness of pancreatic cancer (ZHAI et al., 2008; CHEN et al., 2008). Increased levels of CACYBP/SIP protein were also found in gastric, colon, nasopharyngeal, osteosarcoma, and melanoma (GHOSH et al., 2011; KILANCZYK et al., 2012; EVANS and CORFE, 2013; GHOSH et al., 2013; ZHU et al., 2014). Among them, some studies have found that the level of CACYBP/SIP in gastric cancer cells is positively correlated with cancer differentiation, and we have also found that the high expression of CACYBP/SIP can inhibit the proliferation and invasion ability of gastric cancer cells and inhibit the growth of tumor cells *in vitro* (NING et al., 2007). In colorectal cancer, a study found that the expression of CACYBP was increased in three different primary CRC cell lines and significantly decreased its adhesion and β-catenin levels, suggesting that CACYBP/SIP may be related to CRC metastasis (GHOSH et al., 2011). In renal cancer, the expression of CACYBP/SIP protein was significantly decreased in renal cancer tissues and renal cancer cell lines (GHOSH et al., 2013).

This study demonstrated that CacyBP expression was increased in bladder cancer tissues and cell lines, and CacyBP may be involved in the regulation of malignant biological behavior of bladder cancer. From our previous RNA-seq data of bladder cancer tissues, we found the mRNA levels of CacyBP were upregulated in bladder cancer tissues compared to normal tissues. Then we used CRIPSR/dCas9-KRAB to knock down CacyBP in T24 and 5,637 bladder cancer cells. We found the expression levels of CacyBP were downregulated after transfected with dCas9-KRAB in T24 and 5,637 bladder cancer cells. Then we examined the cell proliferation and migration ability after CacyBP downregulated through a serious of *in vitro* experiments in T24 and 5,637 bladder cancer cells. The results showed that the proliferation and migration ability of T24 and 5,637 bladder cancer cells was decreased after transfected with dCas9-KRAB and sgRNAs targeted CacyBP. Finally, we performed the caspase3-Elisa assay to examine the apoptosis in T24 and 5,637 bladder cancer cells, we found that downregulation of CacyBP promoted the apoptosis in bladder cancer cells.

Collectively, the expression level of CacyBP is frequently increased in bladder cancer. CRISPR/dCas9-KRAB targeting CacyBP inactivates its expression and suggested its potent oncogenous function in bladder cancer through a serious of *in vitro* experiments. It is hoped that these findings will contribute to a potential therapeutic strategy in bladder cancer.

## Method

### Cell Culture and Plasmid Transfection

Human normal urothelial cells SVHUC-1, bladder cancer cells T24, UMUC3, BIU-87 and 5,637 were cultured in DMEM medium with 10% fetal bovine serum, respectively. The fluid was changed once every 2 days, and the passage rate was 1:4 per week. After all cells were cultured for 48 h, the expression of CacyBP/SIP in cells was detected by RT-qPCR. Then T24 and 5,637 bladder cancer cells are used to transfected with dCas9-KRAB and sgRNA. Transfection was performed according to the instructions: T24 and 5,637 bladder cancer cells with a 1×10^6^ density of/ mL were seeded into a 6-well plate. When 80% of the cells were fused, dCas9-KRAB and gRNAs were transfected into the cells. After 6 h of serum-free culture medium, 10% fetal bovine serum DMEM medium was replaced for further culture for 24 h.

### Cell Scratch Assay

Cells were taken from each group and inoculated in a 6-well plate at a density of 1×10^6^ cells/ mL. When the cells were completely filled, a vertical line was drawn with a 10 µL spear head, and the cells were rinsed with PBS for 3 times. Photographs were taken under an inverted microscope and marked as 0 h pictures. Then, 10% fetal bovine serum DMEM medium was added for further culture for 24 h. Photographs were taken under an inverted microscope and labeled as 24 h images. The Image of 0 h was used as reference, and the software ImageJ was used to analyze the relative migration area of cells. The relative cell migration rate is equal to the migration area of the experimental group divided by the migration area of the control group.

### Transwell Migration Assay

The T24 and 5637 cells suspended in 150 μl serum-free medium (2 × 10^5^ cells/ml) were placed on the upper layer of a cell permeable membrane. Following another 24–48 h incubation, the cells migrated through the membrane were stained with 1% Crystal Violet and counted.

### Cell Counting Kit-8 Assay

Cells with good growth and in logarithmic phase were digested into cell suspension, and 3,000–5,000 cells were inoculated into 96-well plates after counting. Nine parallel wells were set in each group. Cell culture plates were taken out after 24, 48 and 72 h, respectively, and the medium in each well was drained. Then 100 μL of the reaction solution diluted 9:1 with normal medium and CCK-8 was added and put back to the cell incubator for incubation for 1 h. Finally, the absorbance of 450 nm wavelength was obtained by using BioTek microplate analyzer.

### RT-qPCR Assay

48 h after transfection, the cells were collected, the total mRNA was extracted, and the target fragment was amplified after reverse transcription. The PCR reaction conditions were as follows: pre-denaturation at 95°C for 30°s, 95°C for 5°s, 60°C for 20°s, 40 cycles. The relative quantitative analysis was performed using 2-ΔΔCT with GAPDH as internal reference. The experiment was repeated for 3 times. CacyBP forward primer:5’-CTCCCATTACAACGGGCTATAC-3’; reverse primer:5’- GAA​CTG​CCT​TCC​ACA​GAG​ATG-3’; GAPDH forward primer:5’-GGAGCGAGATCCCTCCAAAAT-3’; reverse primer:5’-GGCTGTTGTCATACTTCTCATGG-3’.

## Statistical Analysis

All measurement results were expressed as (mean ± standard deviation). SPSS 19.0 was used for statistical analysis of all research data, and LSD-t test was used for pairwise comparison between measurement data groups. *p* < 0.05 indicated that the difference was statistically significant.

## Data Availability

The original contributions presented in the study are included in the article/Supplementary Material, further inquiries can be directed to the corresponding author.
